# Supplementary material for: Microbeam X-ray diffraction study of lipid structure in stratum corneum of human skin
Source: PLoS One. 2020 May 11;15(5):e0233131. doi: 10.1371/journal.pone.0233131 (PMC7213682; doi:10.1371/journal.pone.0233131)
Supplement: S1 Appendix — (DOCX) [file pone.0233131.s001.docx]

S1 Appendix: Interpretation of the X-ray diffraction

Human skin has wrinkles, sweat glands and hairs. Although skin was mildly stretched in this experiment, it is not ensured to be uniformly extended. Thus, each scan at a different position of a human skin sample produced slightly different X-ray diffraction results. However, in most cases where sweat glands or hairs are not close to the X-ray beam, the differences among scans can be explained by different orientations of the same lipid structure in the SC.

The basic structure of the SC is explained as a brick-and-mortar model where a brick represents a flat corneocyte cell and a mortar intercellular lipid (SFig 1a). The X-ray diffraction is presumed to arise from lipids whose lamellar planes are parallel to the skin surface (SFig 1b). The reason the peaks disappear in deep regions of skin even though the X-ray beam is still passing through the SC can be explained as follows. In reciprocal space, Fourier transform of a lamellar lipid structure is an array of points, but in fact it is an array of disks because of the disorder in lipid structure in the SC. To give rise to intensity in the diffraction pattern, this disk must intersect the Ewald sphere (SFig 1e). To realize this condition, the ideal situation is that the lipid layers are parallel to the X-ray beam. This condition is fulfilled at the top of the folded skin. However, the skin is curved by folding (SFig 1c). This curvature is difficult to measure experimentally, but the skin is almost vertical at about 1 mm from the top. Thus, we simulate the top as a semisphere with a radius of 1 mm (SFig 1c). At 10 μm from the surface, about the middle of the thickness of the SC, the skin surface is tilted towards (or away from) the X-ray beam by about 8 degrees (SFig 1e). Since the half-angle of the diffraction spot at *q*=1.0 nm^-1^ in SFig 2a is about 8 degrees across the meridian, the spots from the tilted surfaces of the SC do not intersect the Ewald sphere in reciprocal space and do not produce a diffraction spot (SFigs 1e, 1f) at this depth. However, the lipid layers in the deeper region of the SC still lie parallel to the beam and diffract X-rays. Their contribution to intensity is large because the intensity is higher when the Ewald sphere intersects the disk closer to its center. When the beam passes well below the SC, the SC on the beam is so tilted that there is no diffraction from lipids. This situation should equally apply to all orders of lamellar diffraction peaks regardless of their *q* positions because both the disk in reciprocal space and its distance from the Ewald sphere become proportionally larger when it is further away in the q-direction from the origin (along the vertical axis in SFig 1f). Thus, relative intensity of the peaks should remain unchanged when the lipid layers are inclined towards the X-ray beam.

For this analysis, the intrinsic width of the diffraction peaks (arcs) across the meridian is important. There are two factors that determine this width. One is the size of the coherent area of the intercellular lipids, which is the size of a region where the lipid molecules form a flat lamellar-like structure between cells. The observed spot size (about 8 degrees in SFig 2a) corresponds to an area of the intercellular lipids of about 43 nm. This is a reasonable size in view of electron micrographs[[1](#_ENREF_1)]. If this coherent area is smaller, the diffraction spot extends across the meridian to form a layer line. The other factor is a variation of tilt of lipid layers within the X-ray beam. A line in a diffraction pattern is created when there are highly ordered structures aligned towards one direction, while an arc is created when the structures are aligned towards different directions. When there are lipid lamellar structures with different tilts, for example as in an uneven skin, the diffraction spots appear as arcs across the meridian. Since the diffraction pattern in SFig 2b seems to be made of arcs rather than lines, it can be concluded that the intrinsic size of the spot is as small as in SFig 2a but the skin is more uneven in the sample of SFig 2b. In such an irregular skin sample, diffraction arcs from the SC are observed even when the beam is passing deep in the skin because the large disk of diffraction peak in reciprocal space intersects the Ewald sphere. In an extreme case, the spatial resolution (depth resolution) is lost.
