# Supplementary material for: Microbeam X-ray diffraction study of lipid structure in stratum corneum of human skin
Source: PLoS One. 2020 May 11;15(5):e0233131. doi: 10.1371/journal.pone.0233131 (PMC7213682; doi:10.1371/journal.pone.0233131)
Supplement: S1 Fig — (a) (top) A schematic view of SC with a “brick-and-mortar” model. The “bricks” (green) are keratynocytes and the “mortar” (orange) is intercellular lipids. (bottom) In the mortar, lipid molecules (black sticks) are arranged across the gap. (b) A flat skin sample (top) is folded (bottom) for X-ray diffraction measurement. SC (blue) is at the top of the fold, which is supported by cellular tissues (grey). The SC is investigated with an X-ray beam (red). (c) X-ray penetration at different depths of the SC. Orange arcs represent the intercellular lipid layers that run approximately parallel to the skin surface. The X-ray beam passes along the lipid layers at the tip of the sample. In the deeper region of the SC, the lipid layers are inclined towards or away from the beam in the areas where the beam enters or leaves the sample, but still parallel in the middle. (d) Principle of X-ray diffraction of a lamellar sample in reciprocal space. The two blue disks represent diffraction spots from lipid molecules arranged with a periodicity of 12.5 nm. The radius of the disk, which increases proportionally towards higher reflection orders, is determined by the size of coherent areas of lipid layers. Red lines are on the surface of the Ewald sphere whose radius is the reciprocal of the X-ray wavelength. Intersection of the sphere with the disk is drawn in red broken line. It is this intersection that gives rise to diffraction. (e) Effect of tilt of a skin sample in reciprocal space. If there were no disorder in the lipid layers (i.e. if the coherent area was infinitely large) the disks in reciprocal space would become points and there would be no intersection with the Ewald sphere, hence no diffraction would be observed. However, in reality, because of the limited size of the coherent area, the disks in reciprocal space intersect the Ewald sphere (left). When the lipid layers are too inclined towards the beam, the intersection is lost and no diffraction is observed. In the present [file pone.0233131.s002.docx]

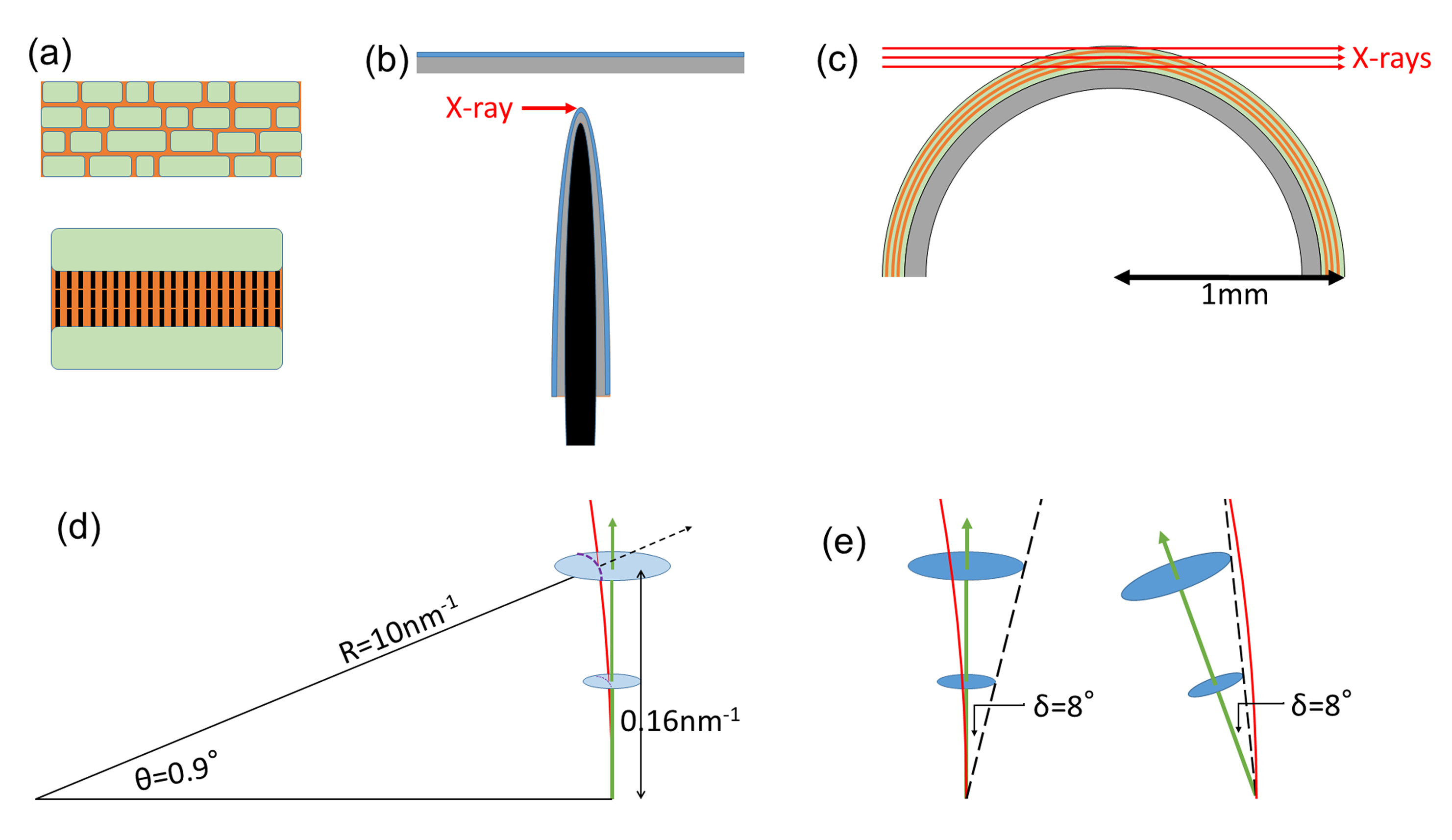


SFigure 1

(a) (top) A schematic view of SC with a “brick-and-mortar” model. The “bricks” (green) are keratynocytes and the “mortar” (orange) is intercellular lipids. (bottom) In the mortar, lipid molecules (black sticks) are arranged across the gap. (b) A flat skin sample (top) is folded (bottom) for X-ray diffraction measurement. SC (blue) is at the top of the fold, which is supported by cellular tissues (grey). The SC is investigated with an X-ray beam (red). (c) X-ray penetration at different depths of the SC. Orange arcs represent the intercellular lipid layers that run approximately parallel to the skin surface. The X-ray beam passes along the lipid layers at the tip of the sample. In the deeper region of the SC, the lipid layers are inclined towards or away from the beam in the areas where the beam enters or leaves the sample, but still parallel in the middle. (d) Principle of X-ray diffraction of a lamellar sample in reciprocal space. The two blue disks represent diffraction spots from lipid molecules arranged with a periodicity of 12.5 nm. The radius of the disk, which increases proportionally towards higher reflection orders, is determined by the size of coherent areas of lipid layers. Red lines are on the surface of the Ewald sphere whose radius is the reciprocal of the X-ray wavelength. Intersection of the sphere with the disk is drawn in red broken line. It is this intersection that gives rise to diffraction. (e) Effect of tilt of a skin sample in reciprocal space. If there were no disorder in the lipid layers (i.e. if the coherent area was infinitely large) the disks in reciprocal space would become points and there would be no intersection with the Ewald sphere, hence no diffraction would be observed. However, in reality, because of the limited size of the coherent area, the disks in reciprocal space intersect the Ewald sphere (left). When the lipid layers are too inclined towards the beam, the intersection is lost and no diffraction is observed. In the present case, since the radius of the disk corresponds to about 8 degrees, no reflection is observed when the lipid layers are tilted more than 8 degrees (right).
