# Supplementary material for: Microbeam X-ray diffraction study of lipid structure in stratum corneum of human skin
Source: PLoS One. 2020 May 11;15(5):e0233131. doi: 10.1371/journal.pone.0233131 (PMC7213682; doi:10.1371/journal.pone.0233131)
Supplement: S2 Fig — (a) A typical X-ray diffraction pattern from human skin at a depth of about 10 μm from the surface of the skin. The sharp spots at q = 1.85 nm-1 in the lower half can be attributed to cholesterol. (b) A diffraction pattern from a more disordered human sample. (DOCX) [file pone.0233131.s003.docx]

(a) (b)







SFigure 2

X-ray diffraction patterns from human skin. (a) A typical X-ray diffraction pattern from human skin at a depth of about 10 µm from the surface of the skin. The sharp spots at *q*=1.85 nm^-1^ in the lower half can be attributed to cholesterol. (b) A diffraction pattern from a more disordered human sample.
